# Impact of Nanoparticles on Male Fertility: What Do We Really Know? A Systematic Review

**DOI:** 10.3390/ijms24010576

**Published:** 2022-12-29

**Authors:** Jean-Philippe Klein, Lionel Mery, Delphine Boudard, Célia Ravel, Michèle Cottier, Dimitrios Bitounis

**Affiliations:** 1 Université Jean Monnet Saint-Étienne, INSERM, SAINBIOSE U1059, F-42023 Saint-Etienne, France; 2 CHU de Saint-Etienne, Service D’Histologie-Embryologie-Cytogénétique, F-42023 Saint-Etienne, France; 3 CHU Rennes, Service de Biologie de la Reproduction-CECOS, F-35000 Rennes, France; 4 Univ Rennes, Inserm, EHESP, IRSET (Institut de Recherche en Santé, Environnement et Travail)—UMR_S 1085, F-35000 Rennes, France

**Keywords:** nanosized objects, testicular biodistribution, reprotoxicity, blood–testis barrier

## Abstract

The real impact of nanoparticles on male fertility is evaluated after a careful analysis of the available literature. The first part reviews animal models to understand the testicular biodistribution and biopersistence of nanoparticles, while the second part evaluates their in vitro and in vivo biotoxicity. Our main findings suggest that nanoparticles are generally able to reach the testicle in small quantities where they persist for several months, regardless of the route of exposure. However, there is not enough evidence that they can cross the blood–testis barrier. Of note, the majority of nanoparticles have low direct toxicity to the testis, but there are indications that some might act as endocrine disruptors. Overall, the impact on spermatogenesis in adults is generally weak and reversible, but exceptions exist and merit increased attention. Finally, we comment on several methodological or analytical biases which have led some studies to exaggerate the reprotoxicity of nanoparticles. In the future, rigorous clinical studies in tandem with mechanistic studies are needed to elucidate the real risk posed by nanoparticles on male fertility.

## 1. Introduction

Since the seminal studies of Carlsen et al. and Swan et al., concerns have been raised in the scientific community about male fertility and particularly sperm number decrease. [1,2] While these observations have not all been confirmed by subsequent studies and regional variability has been highlighted [3,4,5], most studies do describe some sort of decrease in sperm quality [6,7,8,9,10], which has also been recently confirmed by a large meta-analysis [11] focused on western countries. Under the light of available data, it is reasonable to investigate the causes of this degradation and attempt to explain the observed interregional variations.

Decreasing sperm quality seems to be the result of environmental and lifestyle factors, some of which have already been identified: tobacco [12] (particularly intrauterine tobacco exposure [13,14]), heavy metals, such as mercury or lead, [15] heat, [16] and medical treatments, including ionizing radiation and chemotherapeutic agents, all exert direct toxicity on germ cells and adversely impact spermatogenesis. Other agents act by altering the function of the endocrine system and are thus called “endocrine disrupters”. Most notorious among them are persistent organic pollutants (dioxins, furans, etc.), chemicals used in the plastics industry (bisphenol A, phthalates, etc.), pesticides [17,18] (dibromochloro-propane, chlordecone, etc.), and phytoestrogens, such as isoflavones in soy-based preparations [19,20,21]. At the same time, the rise of nanotechnologies over the last three decades has led to an increase in human exposure to engineered nanoparticles. The concomitant increase in human exposure to nanoparticles and decrease in sperm quality has prompted many regulatory bodies to encourage studies on the impact of nanoparticles on human reproduction and particularly on male fertility [22].

Unlike other previous reviews, we chose not to group our results according to the physicochemical characteristics of the particles and particularly their elemental composition [23,24,25]. We made that choice because, even if there is no intrinsic toxicity tied to the nanoscale but rather variable toxicities depending on the considered nanoparticle [26], it is not certain that the elementary composition is the physico-chemical characteristic that has the greatest impact on nanoparticle reprotoxicity. Indeed, it has already been shown several times that other intrinsic characteristics of nanoparticles impact their toxicity (e.g., size, surface area, shape, structure, aggregation, and agglomeration capacity) [27]. In addition, we wanted to start from reproductive physiology and see how it could be disturbed by the nanoparticles rather than starting from the nanoparticles themselves, in order to give a different perspective on these questions.

This review will be divided into two distinct parts. The first one will attempt to synthesize the studies that have worked on testicular biodistribution and biopersistence of nanoparticles. Indeed, because the testis is responsible for spermatogenesis and testosterone production, direct nanoparticle reprotoxicity can only be considered if they are able to reach the testis. In this chapter, the determinants of such biodistribution will be studied in greater detail, be it the physico-chemical characteristics of the particles or the routes of exposure. Special attention will be paid to the interactions between nanoparticles and the blood–testis barrier. The second part of this review will be devoted to the study of the biotoxicity of nanoparticles on cell and animal models through the analysis of the alteration of the histology of the testis, genotoxicity and metabolic damage, disturbance of endocrine system and alteration of spermatogenesis.

## 2. Material and Methods

**Figure 1:**
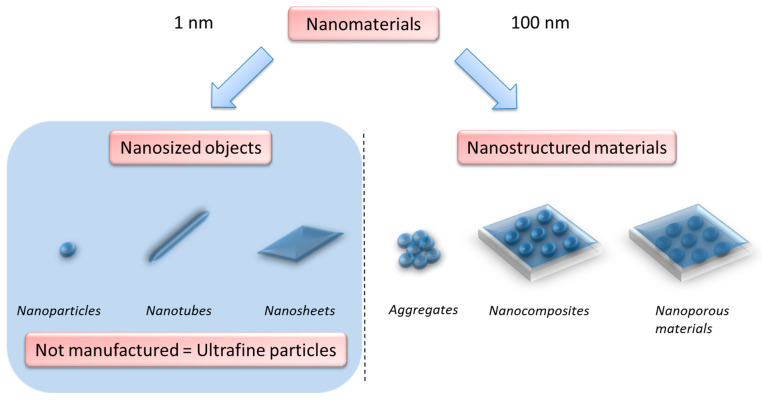
Classification of nanomaterials based on the work of InRS (French Institute for Research and Security). The blue background shows particles on which this review has focused on.

In order to fully understand the spectrum of publications analyzed in this review, we need to define what is meant by the term “nanoparticles”. The large family of nanomaterials as defined by the European Commission and the ISO TS 80004-1 standard covers all objects with an internal or external dimension at the nanoscale, that is to say between 1 and 100 nm [28,29]. On one hand, there belong nanostructured materials, such as aggregates, nanocomposites and nanoporous materials; on the other hand, there are nanosized objects, such as nanosheets, nanotubes and nanoparticles depending on whether one, two or three of their external dimensions ranges between 1 and 100 nm, respectively. When these nano-objects are naturally generated or unintentional by-products of human activity, they are sometimes referred to as ultrafine particles. This review has attempted to analyze all the literature on the action of nano-objects on male reproductive function, including ultrafine particles. For practical reasons, we have grouped here all of these particles under the term “nanoparticles” (Figure 1).

In order to conduct the most exhaustive and transparent review possible, we have followed the reporting guidance for systematic reviews of clinical trials in the health field: the PRISMA statement [30,31]. We searched the Medline database through the Pubmed search engine using the following search equation (last query on 5 May 2020, without any limit):

((“Nanostructures” [Mesh] OR “nanoparticles” [tiab] OR “nanoparticle” [tiab] OR “nanoparticulate” [tiab] OR “nanotube” [tiab] OR “nanotubes” [tiab] OR “nano-sized” [tiab] OR “nanorod” [tiab] OR “nanorods” [tiab]) AND (“Fertility” [Mesh] OR “Infertility, Male” [Mesh] OR “spermiotoxicity” [tiab] OR “reproductive disease “[tiab] OR “ reproductive toxicity” [tiab] OR “testosterone” [MeSH Terms] OR “testosterone” [tiab] OR “testis” [MeSH Terms] OR “testis” [tiab] OR “spermatozoa” [MeSH Terms] OR “spermatozoa” [tiab] OR “sperm” [tiab] OR “semen” [MeSH Terms] OR “semen” [tiab] OR “leydig cells” [MeSH Terms] OR “leydig” [tiab] OR “sertoli cells” [MeSH Terms] OR “sertoli” [tiab] OR “germ cells” [MeSH Terms] OR “germ cells” [tiab] OR “germ cell” [tiab] OR “Semen Analysis” [Mesh] OR “Spermatogenesis” [Mesh] OR “Spermatogenesis” [tiab])) OR ((“Tissue Distribution” [Mesh] OR “biodistribution” [tiab] OR “distribution” [tiab]) AND (“nanostructures” [Mesh] OR “nanoparticles” [tiab] OR “nanoparticle” [tiab] OR “nanoparticulate” [tiab] OR “nanotube” [tiab] OR “nanotubes” [tiab] OR “nano-sized” [tiab] OR “nanorod” [tiab] OR “nanorods” [tiab]) AND (“testis” [MeSH Terms] OR “testis” [tiab]))

### 2.1. Inclusion and Exclusion Criteria

From the identified studies, we collected all the publications that evaluated the distribution, biopersistence, or biotoxicity of nanoparticles on the male reproductive system. Screening excluded studies published in a language other than English or French, studies that did not address nanoparticles as defined above, studies that were not interested in the reproductive system, non-mammalian studies, studies focusing exclusively on the female reproductive system, as well as studies focusing on the reprotoxicity of a combination of transport nanoparticles (for example, liposomes) and reprotoxic molecule. It is also important to note that only studies that addressed male adult or prepubertal male reprotoxicity were integrated. Although the toxicity of nanoparticles on the reproductive organs of the fetus has also been studied in a number of studies, the mechanisms at work in this case are very different from what happens in adults and will not be the subject of this review [32,33,34]. Two authors searched the literature, screened, and selected the final articles to be included. Any discrepancies in choice of articles included in the review were resolved by consensus between the two authors.

### 2.2. Quality and Bias Assessment

The quality of the various articles and the possible biases present were evaluated through several points: good characterization of the particles, relevance of the methods used, rigor of their application, quality of the images provided, likelihood of the results given the physiology of the male reproductive system, honesty in results interpretation, and overall quality of the manuscript. When the biases found were large enough to skew the results of a study, it was excluded after concerted agreement by at least two review authors.

## 3. Results

### 3.1. Search Results and Characteristics of the Included Studies

**Figure 2:**
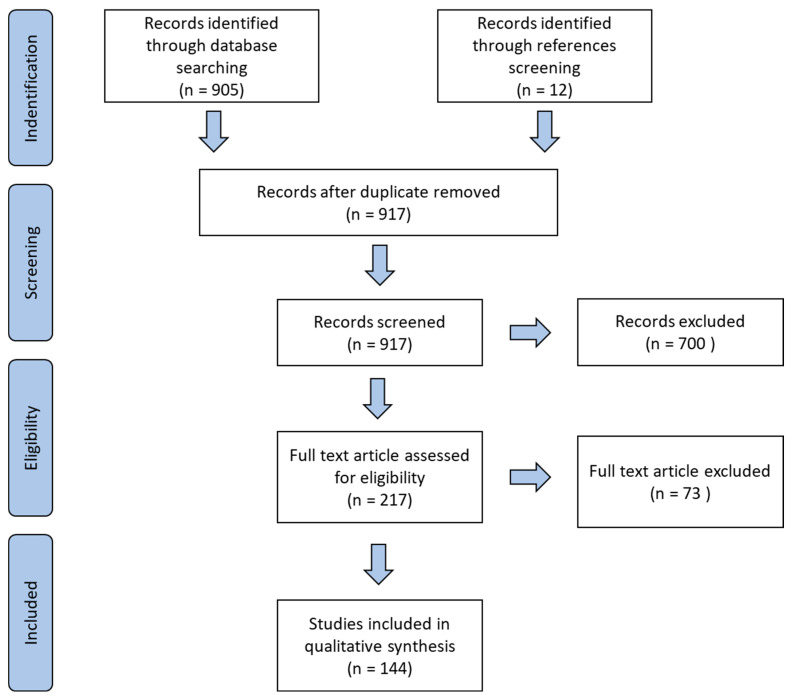
The PRISMA diagram details the search and selection process applied during the review.

A total of 917 articles were identified from our PubMed database search. After screening the titles and abstracts of these articles, 217 were determined eligible for full text review (Nine studies were excluded solely on the basis of language). A total of 73 articles were excluded after full text review based on quality evaluation [35,36,37,38,39,40,41,42,43,44,45,46,47,48,49,50,51,52,53,54,55,56,57,58,59,60,61,62,63,64,65,66,67,68,69,70,71,72,73,74,75,76,77,78,79,80,81,82,83,84,85,86,87,88,89,90,91,92,93,94,95,96,97,98,99,100,101,102,103,104,105,106,107]. Our review is based on the data gathered from the 144 remaining articles (Figure 2).

### 3.2. Testicular Biodistribution and Biopersistence of Nanoparticles

The first point that emerges from the literature on biodistribution of nanoparticles (mostly on rat or mouse models), is that nanoparticles are indeed able to reach the testis. Although publication bias cannot be ruled out (i.e., propensity of the scientific community to publish results that only report on this distribution), it seems legitimate to say that in most cases, nanoparticles are capable of reaching the testis in exposed mammals. Having said that, it is important to first review the body of literature that suggests that NP are indeed capable to reach and cross the blood–testis barrier thus leading to testicular biodistribution of NP. All data collected from studies on the biodistribution of nanoparticles have been grouped in Table 1.

#### Quantification of Testicular Distribution and Biopersistence of Nanoparticles

The overarching theme of the available literature is that the testicle is not a preferred organ for the distribution of nanoparticles regardless the duration of exposure, type of particle, or applied methodology.

Indeed, Balasubramanian et al. show that upon intravenous administration of nanoparticles in rats at concentrations expected to be brought on in an occupational setting, the concentrations of particles observed in the testicle are smaller than for all the other organs [114]. Similarly, most other studies that have made comparisons with other organs also conclude that the testis is not a preferred organ of nanoparticles biodistribution [109,112,115,118,132,135,140,143,145]. Even more so, studies of Miura et al., Choi et al. and Tang et al., Nakkala et al., Leclerc et al. and Liang et al. did not find any testicular distribution of injected titanium oxide, zinc oxide, silver, gold and silica nanoparticles, or orally administrated silica nanoparticles, respectively [125,126,136,138,139,142]; however, in most of these studies, analyses were performed by ICP-AES (inductively coupled plasma atomic emission spectroscopy), a less sensitive technique than ICP-MS (inductively coupled plasma mass spectrometry) used in most other studies. In the end, only Wang et al. reported a predominant distribution of particles to the testis [122].

Beyond the assessment of biodistribution, eight studies tracked the presence of particles in the testis over prolonged periods (up to four months) using ICP-MS or AAS (atomic absorption spectroscopy). Results were conflicting as three of them reported a significant biopersistence of the constituent elements (mostly gold and silver) of the particles within the testis at levels superior to other tested organs [117,120,129]; the other five studies only found a fraction of the particles that initially reached the testis a few months after the end of exposure [121,124,127,134,140]. Nevertheless, all studies found that the particles had not been completely eliminated from the testis, which could lead to accumulation phenomena during periods of prolonged exposure. Some authors justify the strong testicular biopersistence of particles by the presence of the blood–testis barrier which would protect them from cells normally responsible for their elimination, a similar phenomenon to what is observed in the brain. However, to support this mechanistic explanation, the nanoparticles’ ability to cross the blood–testis barrier, as discussed later, should be better understood in future studies.

Wherever possible, we evaluated the proportion of nanoparticles found in the testicle compared to the number of injected particles. This proportion ranged between 1 particle for 10^2^ and 1 in 10^7^, with an average of about 1 in 10^5^. This proportion remains low in comparison with organs such as the liver or the lungs [109,114]. Nevertheless, variations can be important from one study to another and numerous parameters could explain these differences, as is presented in the next section.

### 3.3. Determinant Factors of Testicular Distribution and Biopersistence of Nanoparticles

The testicular distribution and biopersistence of nanoparticles are expected to be contingent on multiple factors, including the employed animal model, the particles’ physicochemical characteristics (size, shape, surface chemistry, tendency to agglomeration), route and duration of exposure, the diluent used at the time of administration [108]. Unfortunately, some of these parameters have been insufficiently studied to allow for solid conclusions about their impact on testicular biodistribution of nanoparticles. Nevertheless, the size of nanoparticles, their surface charge, and the route and duration of exposure have been adequately studied and shown to invariably affect the biological profile of nanoparticles in the testes.

#### 3.3.1. Nanoparticle Size

These studies show that the smaller the particles, the easier they penetrate the testis. Indeed, De Jong et al. show that only 10 nm gold particles are able to reach the testis after intravenous injection. Under identical conditions, gold particles of 50, 100, and 250 nm do not reach it, or in quantities below the detection threshold of ICP-MS [109]. Similarly, after an intravenous injection, Morishita et al. observed in the testis only silica nanoparticles measuring 70 nm in diameter, while those at 300 nm were not found [116]. In a study by Bai et al., after intraperitoneal exposure to 50 nm, 80 nm and 200 nm tin sulfide nanoflowers, tin was detected in testis only for 50 nm and 80 nm particles, despite the observation of the agglomeration of the smallest nanoflowers [137]. Similarly, in a study by Park et al., after oral exposure to 22 nm, 42 nm, 71 nm and 323 nm silver nanoparticles, silver was detected in the testis only for the smallest two [111]. This difference is not clearly reproduced by Lankveld et al. who compared the testicular distribution of silver nanoparticles of 20 nm, 80 nm and 110 nm after intravenous injection: all size groups were detected at roughly comparable concentrations [112]. The same was observed in rats exposed by inhalation to industrial cerium oxide particles in the study by Geraets et al., despite very different sizes (from 10 nm to 5000 nm) [115]. The authors suggested that this is due to the agglomeration phenomena that are more important for the smaller particles, thus making them behave similarly to larger particles. The inter-comparison of these studies is more delicate, as parameters other than the size of nanoparticles are likely to influence their testicular distribution.

#### 3.3.2. Nanoparticles Surface Charge

The studies of Lee et al., Li et al. and Wang et al. analyzed the impact of nanoparticle charge on their testicular biodistribution [119,127,133]. Three to 15 nm gold particles coated with PEG groups with a neutral surface charge had a tendency to remain in the bloodstream and were therefore present in the testicle, a richly vascularized organ, in a fairly large quantity just after the intravenous injection. It seems, however, that they accumulated weakly over time, unlike charged particles (positively or negatively), whose testicular concentration increased more rapidly over time.

#### 3.3.3. Route and Duration of Exposure

The majority of studies have focused on intravenous administration of nanoparticles, probably because of the ease with which it can be implemented and its interest in evaluating the medical applications of nanoparticles. Exposure route is likely to have an impact on testicular biodistribution as different exposure routes may result in a change in nanoparticles corona that is known to modify their ability to travel within the body [148]. However, few studies have compared different exposure pathways between them.

This was the case of the study by Liang et al. which showed that intravenously injected nanoparticles were distributed in the liver, the spleen and the lungs in greater quantity than those injected intraperitoneally. However, their testicular distributions were very weak and did not differ significantly, regardless of the route of injection [128].

In another study, an interesting phenomenon was observed upon intraperitoneal injection of ultra-small gold nanoclusters (size: 3 nm) into mice [129]: during the first 30 days after the injection, nanoparticles seemed to accumulate in the muscle, sparing the testicle. Then, within two months, a blood release took place accompanied by an accumulation of these nanoparticles in various peripheral organs including the testis. It is interesting to note that the same phenomenon was not observed in the study by Balasubramanian et al., upon intravenous injection of 20 nm gold nanoparticles [114]. Further studies will be needed to clarify whether this is related to the intraperitoneal injection route or whether other factors such as physicochemical characteristics of the particles are involved. However, if such a mechanism is confirmed, it could accentuate the testicular biopersistence of nanoparticles and promote their accumulation over extended periods.

The respiratory route has been studied by the team of Geraets et al. who found a relatively weak testicular distribution compared with other organs [115].

Exposure to nanoparticles through the intramuscular route has only been studied by our team using a mouse model. In a first study, we showed a very weak testicular distribution of 450 nm particles (≈1 in 1/10^8^) [149]. In a following study, none of the 70 nm particles that had been injected were found in the testes at the detection thresholds of the techniques used (≈1 in 1/10^5^) [126].

Intra-articular exposure, which mimics the degradation of metal prostheses and the release of debris into the joint fluid, has only been studied by Wang et al. and showed a relatively large distribution of chromium and cobalt nanoparticles to the testis [123]. The chronic nature of the exposure may be partly responsible for these observations.

The majority of studies on the biodistribution of nanoparticles after oral exposure have focused on prolonged exposure times (from 14 days to 90 days) and most of them have used silver nanoparticles [110,111,117,120,135]. Overall, results are fairly consistent in terms of the amount of silver reaching the testis. Among the reviewed articles, two propose an interesting theory about a possible mechanism of testicular biodistribution of nanoparticles after oral exposure. In the study by van der Zande et al., rats were exposed to silver nanoparticles or dissolved silver in the form of silver nitrate for 28 days [117]. The authors showed that about 7% of the particles’ mass solubilized and that only this soluble fraction was absorbed by the stomach. Single particle ICP-MS (SP ICP-MS) analysis revealed the presence of particles in most organs, especially in the testis, even in rats only exposed to soluble silver. The authors hypothesized that particles were not absorbed by the stomach but were rather dissolved and absorbed in soluble form. Then, the solubilized elements at sufficiently high concentrations had the possibility to precipitate in particulate form. This mechanism could be responsible for the strong nanoparticles’ biopersistence in the testicle found. In the study by Lee et al. where silver nanoparticles of 10 nm and 25 nm were used, the authors did not find any effect of particles size [120]. They justified this by the same hypothesis formulated by Zande et al. [150]. However, this hypothesis is not mentioned in the other studies.

It is interesting to note that upon oral exposure of rats to zinc nanoparticles, Choi et al. did not detect testicular biodistribution of particles or accumulation of zinc to any other organ, irrespective of dosage [125]. In contrast, an increase in zinc concentration was observed in the liver, spleen, kidneys and lungs after intravenous exposure. This study is one of the few to compare two routes of exposure and highlights the importance of nanoparticle distribution to several organs, even if in this specific case the testis did not seem to be concerned.

Regarding the duration of exposure, two studies compared the impact of repeated exposures compared to a single exposure. The study by Geraets et al. noted that an increase from 1 day to 28 days of exposure through inhalation favors testicular accumulation of a specific type of cerium oxide particles [115]. Bai et al. found similar results by comparing a single injection of carbon nanotubes to repeated injections over 5 days [113]. Thus, we could conclude that chronic exposure could promote the accumulation of some nanoparticles in the testis. However, it should be noted that in both studies, the cumulative dose for repeated exposures was much higher than the one for single exposure, which alone could explain the spotted differences.

#### 3.3.4. Nanoparticles Interfacing with the Blood–Testis Barrier

**Figure 3:**
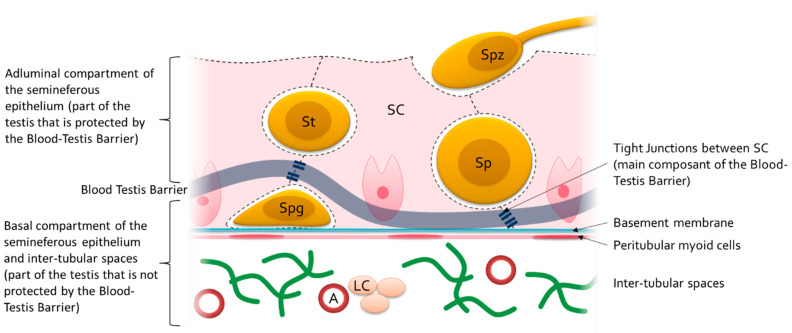
Diagram of the seminiferous epithelium showing the position of the blood–testis barrier (dark blue). Spg: spermatogonia; Sp: spermatocyte; St: spermatid; Spz: spermatozoon; SC: Sertoli cell; LC: Leydig cell; A: arteriole.

The blood–testis barrier is a physiological barrier that separates the seminiferous tube into two compartments: basal and adluminal. Its principal components are the tight junctions formed between the cytoplasmic prolongations of Sertoli cells and its main function is to protect the maturing germinal cells from possible autoimmune reactions. Thus, there are two compartments within the testis: a compartment protected by the blood–testis barrier (the adluminal part of the seminiferous tubes) and a compartment not protected by the blood–testis barrier (the basal part of the seminiferous tubes and the inter-tubular spaces). See Figure 3 for more details. A good knowledge of the interactions between this barrier and nanoparticles is a key issue for the evaluation of their reprotoxicity.

At the time of writing, numerous studies have already suggested that nanoparticles are able to cross the blood–testis barrier and this narrative is adopted by a significant portion of the scientific community [24,25,151,152,153,154,155,156]. However, the majority of the studies that have stated this fact present important methodological biases, rendering their conclusions unreliable [35,40]. This is particularly the case with a study by Kim et al., in which the authors have mistaken testicular autofluorescence, especially fluorescence of spermatozoa head, with nanoparticles-generated fluorescence [38]. We have shown in previous work that testicular autofluorescence was important and should be taken into account when carrying out such studies, otherwise the obtained results may be incorrect [149]. The study by Reyes-Esparza et al. has taken into account the testicular autofluorescence, but the resolution of the confocal microscopy images presented in their articles is insufficient to conclude that the blood–testis barrier has been crossed [130].

The same is true for Wang et al. who only used a global quantification method for the analysis of nanoparticles testicular distribution (i.e., ICP-MS) and cannot thus conclude that the blood–testis barrier has been crossed [122]. Indeed, the blood–testis barrier does not protect the entire testicular parenchyma, so while accumulation of nanoparticles is likely to occur in these unprotected areas, this does not infer a crossing of the blood–testis barrier, as we have shown in our previously cited study [149]. Specifically, the injected particles tend to deposit in the sub-albuginea spaces and inter-tubular spaces, both being unprotected by the blood–testis barrier. An identical confusion is made in the review of Lan and Yang [24]. They describe an interesting theoretical model of nanoparticles crossing the blood–testis barrier but it is based on the conclusions of the study of Park et al. [111]. However, Park et al. cannot conclude that their silver nanoparticles cross the blood–testis barrier as they have, similar to Wang et al., simply measured the total amount of silver within the testicle by ICP-MS.

The study by Li et al. provides rather convincing images of such a crossing which takes place after intra-testicular injection of the nanoparticles [119]. However, this methodological choice, not only is not representative of any route of exposure, but also introduces high risks of particle contamination of the seminiferous tubes at the time of injection and limits the significance of their results.

The study by Snow-Lisy et al. also showed a crossing of the blood–testis barrier. However, their particles benefited from either anatomical targeting—injection into the testicular artery—or biochemical targeting with FSH coating of particles. They also did not check for the absence of interference related to testicular autofluorescence [131].

In conclusion, only two studies provide convincing evidence of this crossing. First, Moroshita et al. studied the testicular biodistribution of intravenously injected 70 nm silica nanoparticles [116]. In this study the observations were made by transmission electron microscopy and nanoparticles were found in the cytoplasm and nucleus of spermatocytes, in the cytoplasm of Sertoli cells, and close to the sperms, attesting a crossing of the blood–testis barrier. Nevertheless, it should be noted that the contrast of silica is not very high in electron microscopy. Second, Kielbik et al. showed the accumulation of fluorescent 50 nm europium zinc oxide nanoparticles in the adluminal part of the seminiferous tubes by scanning fluorimetry after oral exposure in mice attesting to a crossing of blood–testis barrier [146].

Thus, with only a few studies having actually observed a crossing of the blood–testis barrier by nanoparticles under realistic conditions, it is difficult to conclude that nanoparticles in general can cross this barrier. Even if nanoparticles have been convincingly shown to cross other barriers, new studies are still needed to confirm the hypothesis that they are also able to cross the blood–testis barrier.

### 3.4. Biotoxicity

#### 3.4.1. Mature Sperm Model (Ex Vivo Studies)

A number of studies have investigated the action of nanoparticles on mature spermatozoa. For many of them, the doses of nanoparticles put in contact with spermatozoa are immense and have nothing to do with any biological reality [157,158,159,160].

In fact, the two most comprehensive studies on the subject found very little impact of gold and silver nanoparticles on mature spermatozoa, even at significant doses (10 μg/mL) [161,162]. At most, a moderate fixation of some nanoparticles on the thiol groups of the sperm surface membrane was allegedly responsible for a loss of mobility and a steric hindrance which would then limit the fertilizing power of the spermatozoa as shown by Taylor et al. A reduction of sperm mobility was also found after direct exposure of mature spermatozoa to carbon nanotubes [163]. However, in this case, the involved mechanism may be an increase in reactive oxygen species generation [164]. Caldeira et al. did not find any uptake or impact on bull sperm mobility or integrity of DMSA-coated maghemite nanoparticles, even at significant doses (60 μg/mL) [165].

Conversely, the Yoisungnern et al. study found greater toxicity of their silver nanoparticles on mouse spermatozoa, with evidence of particle penetration into spermatozoa cytoplasm, impaired mitochondrial function, decreased fertilizing ability, and impaired embryonic development at relatively low doses (from 1 μg/mL) [166]. Another study conducted by Akhavan et al. observed a massive negative impact on all sperm parameters after exposing mouse spermatozoa to nanocrystals of cadmium tellurium (quantum dots), at relatively low doses too (from 1 μg/mL) [167]. Nevertheless, these direct toxicities must be confirmed by other studies, as they seem very important and quite superior from what has been observed in other studies.

Several other studies measured sperm DNA fragmentation after exposure to titanium dioxide, zinc oxide, silver, cerium dioxide nanoparticles, carbon nanotubes or lipid-core nanocapsules [168,169,170,171,172]. They showed an increase in sperm DNA fragmentation for very small doses of titanium dioxide nanoparticles (1 μg/L) or for very small doses of cerium dioxide nanoparticles (10 μg/L) which could be partially due to oxidative stress [173,174]. In the case of cerium dioxide nanoparticles, a decrease in the fertilizing capacity of the spermatozoa was observed which could either be secondary to this sperm DNA alteration or related to a steric hindrance on the surface of the sperm membrane, as described by Taylor et al. It should be noted that the toxicity of these cerium dioxide particles seems to follow a reverse dose response curve, which means that the lower the dose of particles, the greater the toxicity, which is quite worrisome and should prompt authors to study lower particle concentrations. Conversely, lipid-core nanocapsules, silver nanoparticles and carbon nanotubes do not seem to increase sperm DNA fragmentation even at high doses despite their ability to penetrate cells.

In the end, it is legitimate to question the relevance of a mature sperm model in the context of reprotoxicity studies. Indeed, spermatozoon is a quiescent cell whose DNA is protected by a specific compaction system. It is therefore likely that its resistance to physical or chemical agents is higher than that of a differentiating germ cell. It should be added that the exposure time of a mature spermatozoon to nanoparticles is limited compared to other testicular cells because of its short life span (a few days). In addition, spermatozoa are protected by the blood–testis barrier, and we have seen that it is not yet clearly proven that nanoparticles are able to cross it. For all these reasons, future studies should preferably be designed with a testicular cell line rather than mature sperm cells.

#### 3.4.2. Other Cell Models (In Vitro Studies)

Nanoparticle toxicity has been studied on three testicular cell lines models as presented in a small number of available works.

On different germ cell models, it has been shown that the chemical composition of the particles, as well as their size, can govern their toxicity: small silver particles are particularly toxic [175,176,177,178], while gold nano-sticks show no toxicity [179]. In addition, at identical elemental concentration, particle toxicity appears greater than the toxicity of the constitutive element of these particles in soluble form. This toxicity generally manifests as a decrease in cell proliferation or an increase in cell apoptosis and necrosis [180]. This may be related to interference in intracellular signaling pathways [176], to the induction of cellular autophagy [181], or to impairment of mitochondrial function [182,183]. Disruption of the arrangement of cytoskeleton or nucleoskeleton after exposure to high dose of titanium dioxide nanoparticles or zinc oxide nanoparticles has also been reported [184,185].

On a model of Sertoli cells (TM4 cells), the toxicity of nanoparticles also seems to be related to their chemical composition [186], with silica nanoparticles showing no toxicity [183] and Ag nanoparticles once more being particularly toxic [178,187] even if other particles, especially zinc oxide particles [186], are also able to induce toxicity through oxidative stress [188] and a decrease of mitochondrial potential [189]. At very high doses, gold nanorods seem to be able to interfere with the expression of genes involved in the structure of the blood–testis barrier (occludin, claudin ZO-1 and connexin 43) through an alteration of glycine metabolism [179] or cause aberrant expression of imprinted genes [190]. In a rat Sertoli cell model, high doses of nickel nanoparticles could induce apoptosis by upregulation of specific long non-coding RNA [191].

Finally, studies that looked at Leydig cell models (TM3 cells) showed that some particles were able to limit their proliferation and survival [178,186,187,192,193]. In addition, zinc oxide, diesel and carbon black nanoparticles may have the ability to interfere with testosterone synthesis, possibly through the overexpression of a regulatory gene: the gene regulatory protein spermatogenesis: StAR [192,193].

From these studies, it appears that the lowest dose for a notable cytotoxic effect of nanoparticles remains very high, in the order of 5 to 10 μg/mL; for the alteration of mitochondrial function, the lowest toxic dose is 0.5 µg/mL [182]. It should be noted that such concentrations are almost never found in the testis in biodistribution studies. For example, in the study of Balasubramanian et al., which is among the few that have tried to represent a realistic exposure to nanoparticles, the concentration of nanoparticles in the testis hardly reaches 0.6 ng/mL—a concentration 3–4 orders of magnitude lower than the lowest toxic dose reported in previous cellular model studies. As a result, future studies should lower the administrated nanoparticles doses and extend the exposure times of their models.

#### 3.4.3. In Vivo Studies

Histological anomalies related to particle exposure.

First of all, it must be emphasized that the analysis of histological sections entails a certain degree of subjectivity that can lead to hypothesis confirmation bias. In addition, it requires great expertise. Thus, only publications that presented histological images of sufficient quality to support the described anomalies were included in this review.

Several studies have analyzed testicular parenchymal lesions after exposure to nanoparticles. Most of them first measured the evolution of testicular weight [113,119,123,124,125,127,143,187,194,195,196,197,198,199,200,201,202,203]. It has been shown that, irrespective of the animal model (rats, mice), the particles considered (silver, gold, carbon nanotubes, nano-graphene oxide, carbon black, titanium dioxide, zinc oxide, iron oxide, chromium–cobalt), and the exposure pathways (intravenous, intraperitoneal, inhaled, oral, intra-articular), exposure to nanoparticles do not appear to affect the testicular weight of an adult animal.

Half of the studies analyzed did not report any histological abnormalities after exposure to nanoparticles [119,125,126,127,135,146,197,199,203,204,205,206,207], or did so only for extremely high doses [198,208]. Some studies that have focused on the intravenous exposure to carbon nanotubes [113] or silver nanoparticles [124,195,209], on the intraperitoneal exposure of nano-graphene oxide [201], or on the intra-articular exposure to chromium–cobalt nanoparticles mimicking prosthetic abrasion [123], have observed moderate testicular histological abnormalities: disorganization of the seminiferous epithelium, increased number of apoptotic cells or minor alteration of the Leydig cells. All these abnormalities were shown to be reversible upon cessation of exposure, except for the complete degeneration of some seminiferous tubules after inhalation of carbon black nanoparticles reported by Yoshida et al. [194], a result that was not, however, confirmed in a similar study by Skovmand et al. [203].

In conclusion, the observed histological testicular abnormalities following nanoparticles exposure remain rather limited and most often reversible. Even if these results are reassuring, it should be noted that the histological analysis of the testis is difficult and requires great experience. Guides exist to identify abnormalities that should be investigated when assessing testicular toxicity [210,211]. They especially specify the type of fixation to be used (Bouin liquid, modified Davidson) or to avoid (formalin) as well as the earliest indices of an alteration of spermatogenesis, particularly relying on the analysis of spermatogenetic stages. It seems, however, that most of the work we report here has not followed these recommendations, which weakens our conclusions.

##### Endocrine Disruption

Several studies have hypothesized that nanoparticles could act as endocrine disruptors and have thus tried to evaluate their impact on the synthesis of sex hormones on various animal models.

Many of them have shown that exposure to nanoparticles causes an increase in testosterone synthesis. This is particularly the case with inhaled exposure to diesel particles [212,213,214,215] and carbon black particles [194]. This increase could be secondary to the overexpression of the genes responsible for testosterone synthesis (P450scc and 17β-HSD), as well as regulation genes (StAR), confirming the assumptions made by Komatsu et al. on cell models [192]. Other types of exposures have also resulted in increased testosterone synthesis, including oral or intravenous exposure to titanium dioxide nanoparticles [136,199], inhaled exposure to cellulose nanocrystals [216], intravenous exposure to functionalized gold nanoparticles (PEG-NH2) [119] or to gold nanorods [207], or to oral or intravenous silver nanoparticles [124,217]. The latter could result from an increase in the expression of Cyp11a1 and HSD3B1 genes encoding enzymes involved in steroidogenesis (respectively, P450scc and 3βHSD1) without concomitant increase in the expression of regulatory genes such as StAR, which seems reversible when the exposure stops. The study by Choi and Joo showed that an increase in testosteronemia could also be linked to an inhibition by nanoparticles of its hepatic metabolism via an inhibition of cytochrome P450 (CYP)-mediated testosterone (TST) metabolism [218].

Several studies have found no impact of nanoparticle exposure on testosterone. The particles in question were carbon nanotubes [113], welding fumes particles [219], carbon black or diesel exhaust nanoparticles [203], nanoparticles of silica, [197] titanium dioxide particles [220] and graphene nanosheets [128,201,203] inhaled or injected intravenously, intraperitoneally or intrathecally, or silver nanoparticles ingested during the pubertal period [221,222] or injected during adulthood [223].

Finally, only few studies found a decrease in testosterone synthesis after exposure to nanoparticles. This was an oral exposure to very high doses of titanium dioxide nanoparticles (50 mg/kg/day) which resulted in a decrease in the expression of steroidogenesis enzymes (P450–17α hydroxylase and 17β- HSD) [198] or an oral exposure to very high doses of zinc oxide nanoparticles (150 mg/kg/day) which resulted in a decrease in the expression of StAR [142]. This also was a very prolonged exposure to nickel nanoparticles (15 mg/kg/day for 10 weeks) [224], an intraperitoneal exposure to nanoparticles of uncharacterized molybdenum (10 mg/kg/day for 28 days) [225] or an intraperitoneal exposure to multiwall carbon nanotubes (2 mg/kg/2 days for 30 days) [202]. Lastly, this was a single intravenous exposure to cadmium telluride fluorescent quantum dots (0.2 to 2 nmol) [134] or a single intravenous exposure to silver nanoparticles of 20 or 200 nm (5 to 10 mg/kg) [226]. This last very comprehensive study showed a concomitant elevation of blood LH levels rather evoking a peripheral impairment. However, the moderate changes in steroidogenesis enzymes observed in this study appear to be insufficient to explain the significant fall in male hormones.

In the end, as it has already been done in other reviews [227,228], it is seemingly safe to conclude from the reviewed studies that some nanoparticles have the capacity to act as endocrine disruptors in some specific experimental conditions. This seems particularly true concerning inhaled exposure to carbon particles, such as diesel particles or carbon black particles, even if it has not been observed in all studies [203]. This toxicological mechanism should be the subject of further studies using parallel in vivo and in vitro models which should focus on the physicochemical characteristics of particles which favor this mode of action and its underlying physiological mechanisms. Indeed, the possibility of endocrine disruption via natural exposure routes (oral or inhaled) is worrying because it is likely to affect a large number of people. Moreover, as pointed out by Iavicoli et al., similar to other endocrine disruptors, the effect of nanoparticles does not necessarily follow a conventional dose–response curve [227]. Some studies have even shown a greater effect for lower doses of nanoparticles [136,198,213,215,217,226]. Thus, even moderate environmental exposures could be sufficient to induce a real biological effect.

##### Impact on Spermatogenesis and Capacity to Bear Offspring

First, it is important to understand that an assessment of the impact of nanoparticles on spermatogenesis cannot be done without measuring the daily sperm production or, failing that, an account of the number of spermatozoa in the ejaculate or in the epididymis. Thus, studies that only measured mobility parameters with the CASA system [128,224] or sperm morphology [229,230] do not offer a clear enough vision of the state of spermatogenesis to be included in this review.

Several studies found that nanoparticles did not impact the spermatic parameters [113,124,146,203,220,231], or did so for very high and unrealistic doses [123,137,142,198,200,232,233]. When an impact was found, it was usually reversible once exposure stopped [195,197,201,219,223]. Only Yoshida et al. and Farcas et al. found that inhaled carbon black nanoparticles and inhaled cellulose nanocrystals negatively impacted the spermatogenesis of adult mice, respectively [194,216]. Interestingly, if Alabi et al. did not show any impact of solid lipid nanoparticles or superparamagnetic iron oxide nanoparticles on sperm count, they found that the simultaneous exposure to these two types of particles had a negative impact, suggesting that reprotoxic effect may be potentiated by co-exposure to nanoparticles [234]. It is also interesting to note that the period of life in which exposure occurs may be important to consider in order to assess the impact of nanoparticles on spermatogenesis. Indeed, Sleiman et al. and Mathias et al. found a significant decrease in daily sperm production after chronic oral exposure of prepubertal mice to relatively low doses of silver nanoparticles [217,221,222]. This effect may be related to direct toxicity of silver nanoparticles on sperm cells rather than to an endocrine disruption effect because, in their study, testosterone levels were unaffected for doses where signs of spermatogenesis alteration were observed. A similar result was reported by Zhang et al. in mice but for higher doses [187]. These results are summarized in Table 2.

In addition, Bai et al., Liang et al., Nirmal et al., Li et al., Li et al., Garcia et al., Park et al., Wolterbeek et al., and Patra et al. exposed male mice to carbon nanotubes, graphene nanosheets, functionalized gold nanoparticles (PEG-NH2), silver nanoparticles, iron oxide nanoparticles, synthetic amorphous silica and zinc oxide nanoparticles, respectively, and tested their fertility by mating them with female mice. No differences in sexual behavior, ability to initiate litter, number of pups per litter or offspring state of health were found compared to the control group [113,119,124,128,134,201,231,237]; only the study of Park et al. reported a non-significant alteration in the sex ratio of pups [205].

Finally, inferring that nanoparticles are reprotoxic implies that exposure to nanoparticles affects spermatogenesis and the ability to obtain healthy offspring. Surprisingly enough, only a few studies have looked at these parameters. They predominantly reported a moderate toxicity of nanoparticles on spermatogenesis and the ability to bear offspring, even if the pre-pubertal period seems to be a riskier period. Although these results are generally reassuring, they must be confirmed for particles with different physicochemical characteristics and for varied exposure routes. Future studies on the reprotoxicity of nanoparticles will need to include the evaluation of sperm parameters in their work because they are probably the most relevant markers of the evaluation of the reprotoxicity of nanoparticles.

##### Genotoxicity and Impact on Metabolism of Testicular Cells

The importance of the testicular genome renders genotoxicity studies particularly pertinent. Noxious agents might exert transgenerational impact with unpredictable consequences, as even slight and transient genomic alterations have the potential to increase offspring mortality and morbidity.

Although nanoparticle genotoxicity has already been the subject of many studies [238,239], very few have been performed specifically on germ-line cells. In their study, Yauk et al. compared DNA damages on germ-line cells between two groups of adult male mice. The first group was exposed to air polluted by two steel mills and one highway, while the other one was exposed to the same polluted air after its filtration by a 0.3 μm filter [240]. They observed an increase in persistent genomic abnormalities, including increased frequency of DNA breaks, double-strand mutations, and DNA hypermethylation. Thus, the known genotoxicity of certain nanoparticles on somatic cells could well be at the same level for germ cells. However, it is essential that additional work confirms these suspicions.

A group of studies investigated the ability of nanoparticles to induce testicular oxidative stress in vivo. It has been shown that carbon nanotubes [113,202,236], silica nanoparticles [197] (63 nm), chromium–cobalt nanoparticles [123], tellurium nanorods [241], zinc nanoparticles [141] and titanium dioxide nanoparticles [242] have the ability to transiently increase testicular malondialdehyde (MDA) levels. Catalase and superoxide dismutase activity, enzymes counter-acting oxidative stress, do not seem to be affected by silica nanoparticles, but titanium dioxide nanoparticles seem to increase and decrease catalase and superoxide dismutase activity, respectively. The activities of all the antioxidant enzymes appear to be diminished in the presence of cobalt–chromium nanoparticles [123], tellurium nanorods [241], zinc nanoparticles [141] and nickel nanoparticles [191]. It should be noted that the study of Braydich-Stolle et al. that focused on measuring the production of free radicals on a cellular model in the presence of silver nanoparticles showed only very little prooxidant activity [176], even if an in vivo study showed their ability to reduce testis anti-oxidant capacity at very low doses [147].

In conclusion, some nanoparticles seem to be able to induce oxidative stress or other metabolic abnormalities such as a decrease in testicular ATP synthesis [197] or an overexpression of inflammatory cytokines [216,243]. While it is interesting to explore these different physiological tracks, this should not be an end in itself. The purpose of such studies could be to establish a link between these metabolic alterations and genotoxicity on germinal cells. This was the aim of Asare et al. who intravenously administered silver and titanium nanoparticles to mice [244]. However, despite some signs of genotoxicity (single strand DNA breaks) and overexpression of some antioxidant genes, their results do not clearly confirm this link. This could be due, among other reasons, to the choice of an acute exposure to nanoparticles associated with too short of a follow-up study.

Some studies have attempted to evaluate the impact of nanoparticles directly on the fragmentation of sperm DNA, some kind of final control of their genotoxicity. From one study to another, the results are contradictory (Table 2), even for similar nanoparticles. This is particularly the case between the study from Bai et al. and from Fang et al. which both evaluated multiwall carbon nanotubes reproductive impact; although the route of administration, intravenous and oral, respectively, and especially the administered dose, 5 mg/kg/day and 100 mg/kg/day, respectively, could explain these discrepancies [113,235].

## 4. Discussion

Before answering the question “what do we really know about the impact of nanoparticles on male reproductive function”, we have to consider two possible biases that could undermine the relevance of our analysis and probably that of other systematic reviews on this otherwise very interesting and promising topic [245]. The first one is the publication bias, especially on biodistribution studies. By comparison, it is indeed easier to demonstrate the presence of a particle within an organ such as the testis than proving its complete absence. It is, therefore, possible that certain studies that did not detect any testicular biodistribution of nanoparticles or any impact on the reproductive function was never published. The second one is confirmation bias. While scanning the literature, we could see that certain authors have a tendency to overestimate the real reprotoxicity of nanoparticles, probably in order to facilitate the publication of their work. They reach such conclusions by using extremely high nanoparticle doses, by exaggerating the significance of their results, or, in a few cases, by even falsifying their data. We have strived throughout this analysis in order to identify studies that report on tangible biological data, but it is possible that confirmation bias has still skewed our conclusions. Even if this is the case, this type of bias would steer us toward exaggerating the reprotoxicity of nanoparticles.

Concerning the testicular biodistribution and biopersistence of nanoparticles, we observe that in the majority of cases, they are capable of reaching the testicle, but, most frequently, in very small quantities. The route and duration of exposure are significantly important when it comes to testicular biodistribution, with physiological routes (breathing and eating) leading to smaller levels of biodistribution when compared to parenteral routes. Similarly, acute exposures seem to lead to lower testicular biodistribution than chronic ones.

Among the nanoparticles’ physicochemical characteristics of which the impact has been studied, smaller size and non-neutral surface charge seem to favor their testicular biodistribution and accumulation, respectively. Still, it is not currently possible to claim that most nanoparticles are capable of crossing the hematotesticular barrier. Furthermore, analysis of the literature showed that the quantities of particles that reach the testicles upon acute exposure are probably too weak to induce any toxic phenomena. In fact, the most worrying result comes from observing a possible accumulation of particles inside the testicle, probably due to the organ’s limited ability to eliminate them. In a recent work, Heringa et al. demonstrated the extent to which tissue accumulation of particles during prolonged exposures may increase the risk of long-term testicular toxicity [246].

Therefore, studies which are to be performed in the future on this subject have to focus on prolonged exposures that are more likely to promote important testicular accumulation of nanoparticles. They equally have to compare the impact of other particle physicochemical characteristics, such as their shape, chemical composition, agglomeration tendency, as well as precisely study the interactions between nanoparticles and the hematotesticular barrier by employing co-localization techniques or in vitro barrier models [247]. Additionally, future work should be careful to identify any phenomenon of storage and release of nanoparticles, especially from muscle and liver, by analyzing content of these organs in parallel with the testicle and by performing a prolonged follow-up (at least 90 days) of animals.

Future studies should equally try to emulate more plausible exposure scenarios because, as it was already mentioned, the utilized doses in the majority of toxicological studies are far greater than what could be expected in the context of human exposure under typical circumstances [114,248]. Starting with the exposure limit values established by the different health monitoring bodies could be an elegant solution for choosing the doses of particles tested [203]. For example, the National Institute for Occupational Safety and Health suggested, in 2013, a 1 μg/m^3^ of air limit for carbon nanotubes occupational exposure. Even considering that all the inhaled particles reach the respiratory mucosa, which is false [249], this would lead to a daily dose of 12 μg. Taking into account inter-species extrapolation [250], the daily dose to be tested should not exceed 2 μg/kg/day for mice and 1 μg/kg/day for rats. Unfortunately, the doses used in the studies we report here ranged from 5 to 100 mg/kg/day [113,235].

That said, concerning testicular biotoxicity studies of nanoparticles, they show that the hypothesis of a direct testicular toxicity caused by testicular tissue alteration or at the level of spermatogenesis is rather improbable for the majority of nanoparticles and routes of exposure. Indeed, most studies do not observe any such alterations and those observed are reversible, despite the use of very elevated particle doses. Yet, there appear to be some differences based on the type of nanoparticles. Silver nanoparticles and some nano-crystals (cadmium tellurium, cellulose) have also shown elevated toxicity both in vivo and in vitro when compared to other types of nanoparticles, which suggests that their elemental composition plays a significant role in their toxic potential. It is not impossible, though, that in certain cases toxicity attributed to nanoparticles might in reality be due to leached ions upon their partial dissolution.

Even with the exception of certain types of particles, we can rest assured about the general lack of direct nanoparticle testicular toxicity, but two modes of action are still cause for concern: nanoparticle genotoxicity on germ cells and their ability to induce endocrine disruption. Indeed, quite a few studies have explored these two mechanisms of particle toxicity, even though some have shown that respiratory exposure to diesel or carbon black nanoparticles to doses corresponding to probable human exposure scenarios could have this type of effect.

**Figure 4:**
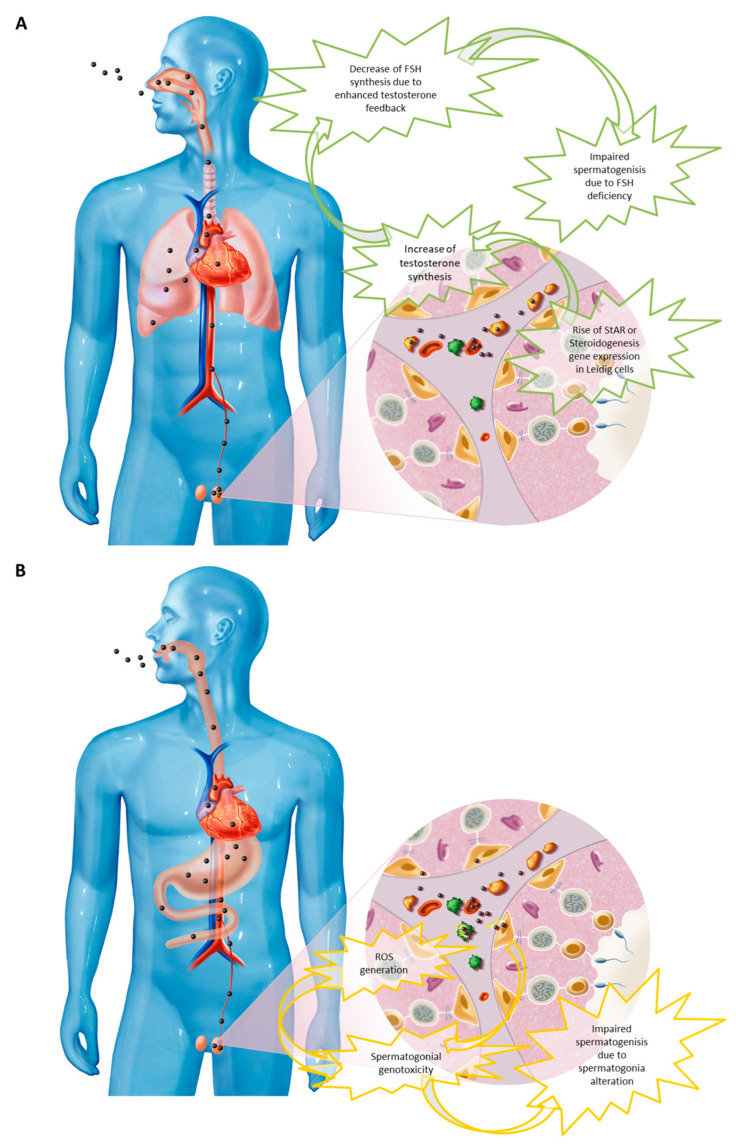
Two plausible scenarios exposure based on gathered data. (**A**) Chronic exposure by inhaled carbon nanoparticles (e.g., diesel particles) able to cross the alveolo–capillary barrier and reach testis inter-tubular spaces through the bloodstream. Increased testosterone synthesis by Leydig cells due to the action of particles on regulating intracellular signaling pathways. Increased negative feedback of testosterone on pituitary follicle stimulating hormone (FSH) synthesis resulting in spermatogenesis inhibition. (**B**) Chronic oral exposure to metallic nanoparticles (e.g., silver nanoparticles) able to cross the digestive barrier and join the inter-tubular spaces and the base of the testicular seminiferous tubes through the bloodstream. Recruitment of local inflammatory cells generating reactive oxygen species (ROS) and direct toxicity on spermatogonia causing spermatogenesis alteration.

Based on the gathered data, some plausible scenarios for nanoparticle-related reprotoxicity incidents in humans would most probably be related to chronic exposure taking place through a natural route—either from food consumption or the respiratory tract—of which the main effectors would be smaller-sized nanoparticles with non-neutral surface charge, able to pass into the bloodstream and reach the testicles. Once inside the testicles, they would persist during a prolonged period of time, probably in the inter-tubular space and in the base of seminiferous tubes. There, they would have the chance to reach the cytoplasm of Leydig cells and perturb hormonal synthesis, specifically the synthesis of testosterone, by interfering with inter-cellular signaling. They could thus be the root cause of a spike or decrease in testosterone levels which, in both cases, would perturb spermatogenesis, either by reduced FSH synthesis because of an intensified negative feedback loop, or by reduced inter-tubular concentration of testosterone. At the same time, the presence of nanoparticles could induce genotoxic effects on spermatogonia (germ cells unprotected by the blood–testis barrier), which in turn could alter the process of spermatogenesis, degrade the germinal genome, lower the chances for embryo implantations, and increase the risk of miscarriage (Figure 4). Another possible mechanism of nanoparticles reprotoxicity not addressed in this review but which should also be explored is in utero exposure toxicity.

To close this literature overview, we have to point out that at the time of writing and to our knowledge, there is no epidemiological study of the possible reprotoxic effects of nanoparticles even though we are faced with ever increasing levels of exposure. This is probably due to the fact that it is difficult to precisely evaluate human exposure to nanoparticles (especially in non-occupational environments) or analyse them in biological samples [251]. However, these studies are an indispensable complement to in vitro experiments and animal models described in this study. These epidemiological studies have to dedicate themselves to determining the real levels of exposure to the most frequently used nanoparticles, the routes of exposure leading to increased particle load of the human organism, and the links between this load and biomarkers of male fertility. To this end, our team has developed an innovative approach to measure the nanoparticle load in the seminal and follicular fluid [252].

That said, it would be naïve to expect the actual cross-testing of all the variations in physicochemical characteristics of nanoparticles with all the modes of human exposure in order to create theoretical models that would allow us to predict the risk of reprotoxic phenomena. Still, we do think that realistic exposure scenarios have to be tested on a case-by-case basis. These works should associate data from animal models (which recreate the exposure case under study) with data from cellular models (so as to mechanistically explain any eventual reprotoxicity) and epidemiological data from the epidemiological monitoring of exposed individuals. For the time being, and despite various existing recommendations, the available studies are very much far from reality.

In conclusion, it seems improbable that all nanoparticles are generally toxic against the male reproductive system. On the contrary, the available information sourced from the literature is generally reassuring. Specific conditions such as prolonged exposure, the potential for genotoxic effects and endocrine disruption exhibited by certain types of particles, or the admittedly higher reprotoxicity of silver nanoparticles merit additional investigation. Still, we have to beware of scaremongering narratives and try to maintain the highest possible scientific rigor in the implementation of future studies. During a period when trust in scientific progress is undermined, it is the duty of the scientific community to intensify its efforts for the optimal interpretation of obtained results.

## 5. Highlights

Engineered nanoparticles have a generally low testicular biodistribution which varies with their size, surface charge, and route of administration. Their reprotoxicity in male adults appears inferior to what a quick glance of the available literature suggests. However, the ability of some nanoparticles to act as endocrine disruptors or express direct genotoxicity on germ cells needs to be evaluated using chronic cellular and animal models.

## Figures and Tables

**Table 1:** Summary of studies exploring testicular biodistribution of nanoparticles. ICP-MS: inductively coupled plasma mass spectrometry, SP-ICP-MS: single particle ICP-MS, ICP-AES: inductively coupled plasma atomic emission spectroscopy, AAS: atomic absorption spectroscopy, TEM: transmission electron microscopy, HPLC: high performance liquid chromatography, FITC: fluorescein isothiocyanate, PEG: poly ethylene glycol, FSH: follicle stimulating hormone.

Publication	Animal Model	Particle Model	Particle Concentration	Route and Duration of Exposure	Particle Measurement Method	Results	Particles Ratio Reaching Testis (Rounded to the Nearest Decimal)
Araujo et al., 1999 [108]	Adult rats.	Polymethyl (2–14 C) methacrylate nanoparticles of a diameter of 130 ± 30 nm in various media to facilitate absorption	2.5 mg per rat	Oral route Single exposure	Radioactivity content of testis measured in a scintillation counter	Testis distribution was maximal in the first hours following exposure. Ratio were slightly modified by the dilution medium.	1/10^5^
De Jong et al., 2008 [109]	Adult rats	Gold nanoparticles Size: 10 nm, 50 nm, 100 nm and 250 nm	From 5.1 × 10^12^ or 80 µg for the 10 nm particles to 3.2 × 10^8^ or 10 µg for 250 nm particles	Intravenous injection Single exposure Measure at 24 h	Gold testis concentration measured by ICP-MS	Testis distribution only observed for 10 nm particles.	1/10^4^
Kim et al., 2010 [110]	Adult rats	Silver nanoparticles Size: 56 nm	30 mg/kg/day 125 mg/kg/day 500 mg/kg/day	Oral route 90 days of exposure	Silver testis concentration measured by AAS	Testis distribution (µg/g wet weight): 6.56 for low dose, 11.84 for medium dose, 23.75 for high dose	Low dose: 1/10^6^
Park et al., 2010 [111]	Adult mice	Silver nanoparticles Size: 22 nm, 42 nm, 71 nm and 323 nm.	1 mg/kg/day 0.25 mg/kg/day, 0.5 mg/kg/day or 1 mg/kg/day for 42 nm particles	Oral route 14 days of exposure (or 28 days for 42 nm particles)	Silver testis concentration measured by ICP-MS	Testis distribution for both 22 nm and 42 nm particles.	Data not available
Lankveld et al., 2010 [112]	Adult rats	Silver nanoparticles Size: 20 nm, 80 nm and 110 nm	20 nm: 23.8 µg/mL 80 nm: 26.4 µg/mL 110 nm: 27.6 µg/mL.	Intravenous injection 1 mL/day for 5 days.	Silver testis concentration measured by ICP-MS	Testis distribution observed for all particles. Max distribution at day 6 for 20 nm particles.	For 20 nm: 1/10^3^
Bai et al., 2010 [113]	Adult mice	64Cu-labeled carboxylated carbon nanotubes	5 mg/kg	Intravenous injection Single exposure or 5 injection over 13 days.	Radioactivity measurement using an automatic γ-counter	Testis distribution at 24 h: 151 ng/g tissue weight.	Single exposure:1/10^3^
Balasubramanian et al., 2010 [114]	Adult rats	Gold nanoparticles Size: 20 nm	0.2 mL with a concentration of 15.1 µg/mL	Intravenous injection Single exposure with measurement from day 1 to 2 months	Gold testis concentration measured by ICP-MS	Low but significant testis distribution at 1 and 2 months.	1/10^4^
Geraets et al., 2012 [115]	Adult rats	Cerium oxide nanoparticles Size: 5–10 nm, 40 nm and 5000 nm	Estimated dose: 5000 nm: 4.24 mg 40 nm: 1.54 mg 5–10 nm: 0.83 mg	Inhalation exposure 6 h/day for 1 day or 28 days.	Cerium testis concentration measured by ICP-MS	Particles distribution lower for testis than for other organs but an accumulation phenomenon is observed over 28 days especially for the largest particles	Single exposure: 1/10^4^
Morishita et al., 2012 [116]	Adult mice	Amorphous nanosilica nanoparticles Size: 70 nm and 300 nm.	0.8 mg per mouse	Intravenous injection 2 consecutive day of injection with measurement at 48 h or one week.	Particles counting in TEM	Testis distribution of 70 nm particles but not of 300 nm particles. Crossing of blood–testis barrier.	Data not available
Van der Zande et al., 2012 [117]	Adult rats	Silver nanoparticles or dissolve AgNO_3_ Size: 15 nm particles coated with polyvinylpyrrolidone and 20 nm non coated particles.	Nanoparticles: 90 mg/kg AgNO_3_: 9 mg/kg	Oral route 4 weeks	Silver testis concentration measured by AAS and particles counting by SP-ICP-MS	Testis distribution of dissolve AgNO_3_ greater than silver nanoparticles. Authors hypothesise that most silver observed in testes after particle exposure comes from silver solubilization.	Data not available
Ye et al., 2012 [118]	Adult male rhesus macaques	Phopholipid-micelle encapsulated cadmium selenium fluorescent quantum dots	25 mg/kg	Intravenous injection Single exposure with measurement at 90 days.	Cadmium testis concentration measured by ICP-MS	Low but significant testis distribution	1/10^5^
Li et al., 2012 [119]	Adult mice	ω-methoxy (mPEG) and ω-aminoethyl (PEG-NH2) poly (ethylene glycol) capped gold nanoparticles. Size 14 nm.	45 mg/kg	Intravenous injection Single exposure	Gold testis concentration measured by ICP-MS	Testis distribution of both nanoparticles	mPEG gold nanoparticles: 1/10^4^ PEH-NH2 gold nanoparticles: 1/10^3^
Lee et al., 2013 [120]	Adult rats	Silver nanoparticles Size: 10 nm and 25 nm	100 mg/kg/day 500 mg/kg/day	Oral route 4 weeks	Silver testis concentration measured by AAS	Testis distribution of both nanoparticles in comparable amount	Both doses and sizes: 1/10^6^
Lee et al., 2013 [121]	Adult rabits	Citrate-coated silver nanoparticles Size: 7.9 nm	5 mg/kg 0.5 mg/kg	Intravenous injection Single exposure with measurement at 1 day, 7 days et 28 days	Silver testis concentration measured by ICP-MS	Testis distribution at each time of analysis. Predominant biliary excretion	Both doses: 1/10^3^
Wang et al., 2013 [122]	Adult mice	Silver nanoparticles Size: 25 nm	1.3 mg/kg/2 days	Intravenous injection 4 weeks exposure. One injection/2 days	Silver testis concentration measured by ICP-MS	Testis distribution of particles	1/10^4^
Wang et al., 2013 [123]	Adult rats	Cobalt–chromium nanoparticles Size: 55 nm	20 µg/kg 100 µg/kg 500 µg/kg	Intraarticular injection 1/week for 10 weeks	Cobalt and chromium testis concentration measured by ICP-MS	Testis distribution of particles higher than observed serum concentration	Low dose: 1/10^3^
Garcia et al., 2014 [124]	Adult mice	Citrate-coated silver nanoparticles Size: 10 nm	1 mg/kg	Intravenous injection 1 injection per 3 days for 12 days (5 injections)	Silver testis concentration measured by ICP-MS	Testis distribution of particles	1/10^5^
Choi et al., 2015 [125]	Adult rats	Zinc oxide nanoparticles. Size:15 nm	30 mg/kg 3 mg/kg	Intravenous injection or oral route. Single exposure	Silver testis concentration measured by ICP-AES after acid mineralization.	No testicular distribution regardless of dose or route of exposure.	Nil
Leclerc et al., 2015 [126]	Adult mice	FITC silica shell gold core particles Size: 70 nm	1.6 × 10^13^ particles/mouse	Intramuscular injection. Single exposure	Silica and gold testis concentration measured by ICP-AES and confocal microscopy	No testicular distribution	Nil
Lee et al., 2015 [127]	Adult mice	Amine or carboxyl or neutral polyethylene glycol-coated gold nanoparticles Size: 15 nm	1 mg/kg	Intravenous injection Single exposure with measurement from 30 min to 6 month after exposure	Gold testis concentration measured by ICP-MS	Early testis distribution for neutral nanoparticles and late testis distribution for charged (amine or carboxyl) nanoparticles.	Amine and carboxyl nanoparticles: 1/10^4^
Liang et al., 2015 [128]	Adult mice	Sheets of nanoscale graphene oxide labeled with ^125^I. Size: 55 nm/4 nm or 240 nm/4 nm	6.25 mg/k to 25 mg/kg (intravenous exposure) 24 mg/kg à 60 mg/kg (intraperitoneal exposure)	Intravenous injection, single exposure Intraperitoneal exposure, one injection per day for 5 days	Radioactivity measurement using a γ-counter	Very low testicular biodistribution regardless of dose, particles size or route of exposure	<1/10^5^
Zhang et al., 2015 [129]	Adult mice	Glutathione-protected gold nanoclusters Size: 3 nm	5.9 mg/kg	Intraperitoneal exposure Single injection with measurement from one to 90 days	Gold testis concentration measured by ICP-MS	Late testicular biodistribution after a period of muscle storage.	1/10^4^
Reyes-Esparza et al., 2015 [130]	Adult rats	Dextrin-coated cadmium sulfide nanoparticles Size: 3 nm	100 µg/kg/day	Intraperitoneal exposure 7 consecutive days	Fluorescence detection of Quantum dots by confocal microscopy	Exogenous fluorescence observed in testes	Data not available
Snow-Lisy et al., 2015 [131]	Adult rats	Poly(lactic-co-glycolic acid) particles (potentially coated with TAT peptide or FSH) Size: 280–315 nm	35 mg/kg	Intravenous or intraarterial exposure	HPLC and fluorescence detection by epifluorescence microscopy	Higher testicular biodistribution for intraarterial injected uncoated particles. Crossing of blood–testis barrier observed.	From 1/10^5^ to 1/10^3^
Creutzenberg et al., 2015 [132]	Adult rats	Europium oxide nanoparticles Size: 80 nm (strong agglomeration)	384.3 µg over 6 h	Inhalation exposure	Europium testis concentration measured by ICP-MS	Testis distribution of particles	1/10^5^
Wang et al., 2016 [133]	Adult mice	Positive or negative or neutral glutathione-protected gold nanoclusters Size: 3 nm	5.9 mg/kg	Intraperitoneal exposure Single injection with measurement from one to 90 days	Gold testis concentration measured by ICP-MS	Biphasic testicular biodistribution, more important for negative particles .	Negative nanoparticles: 1/10^3^
Li et al., 2016 [134]	Adult mice	Cadmium telluride fluorescent quantum dots Size: 2 nm	2 nmol per mouse 0.2 nmol per mouse	Intravenous injection, single exposure with measurement from 3 hours h to 90 days after exposure	Cadmium and telluride testis concentration measured by ICP-MS	High early distribution of quantum dots then progressive elimination over 90 days	1/10^3^ early after exposure
Qin et al., 2016 [135]	Adult rats	Polyvinilpyrrolidone coated silver nanoparticles Size: 30 nm	0.5 mg/kg/day 1 mg/kg/day	Oral exposure 28 consecutive days	Silver testis concentration measured by AAS	Lower testis distribution than the equivalent dose of AgNO_3_	1/10^5^
Miura et al., 2017 [136]	Adult mice	Titanium dioxide nanoparticles Size: 20 nm	0.1 mg/kg/week 1 mg/kg/week 2 mg/kg/week 10 mg/kg/week	Intravenous injection 4 consecutive weeks	Titanium testis concentration measured by ICP-MS	No testicular distribution	Nil
Bai et al., 2018 [137]	Adult mice	Tin sulfide nanoflowers Size: 50 nm, 80 nm and 200 nm	0.38 mg/kg/day 3.8 mg/kg/day 38 mg/kg/week	Intraperitoneal exposure 6 times a week for 4 consecutive weeks	Tin testis concentration measured by ICP-MS	Distribution for 50 and 80 nm particles only at the highest concentration	Between 1/10^4^ and 1/10^5^
Nakkala et al., 2018 [138]	Adult rat	Silver nanoparticles Size (dynamic light scattering): 32 nm	5 mg/kg/day 10 mg/kg/day	Oral exposure 28 consecutive days	Silver testis concentration measured by ICP-AES	No testicular distribution	Nil
Liang et al., 2018 [139]	Adult rat	Silica nanoparticles and microparticles Size: 25 nm and 1 µm, respectively	166.6 mg/kg/day 500 mg/kg/day 1500 mg/kg/day	Oral exposure 90 consecutive days	Silicon testis concentration measured by ICP-AES	No testicular distribution	Nil
Lee et al., 2018 [140]	Adult rat	Silver and/or Gold nanoparticles Size: 14 or 10 nm, respectively	10 µg/kg/day 100 µg/kg/day	Intravenous injection 4 consecutive weeks	Silver or gold testis concentration measured by AAS	Low testicular distribution for silver nanoparticles in comparison to other organs. No testicular distribution for gold nanoparticles	Between 1/10^4^ and 1/10^5^ for silver nanoparticles
Salimi et al., 2019 [141]	Adult mice	Zinc nanoparticles Size: 30 to 80 nm	1 to 5 g/kg/day	Oral exposure 14 consecutive days	Zinc testis concentration measured by AAS	Low testicular distribution	1/10^6^
Tang et al., 2019 [142]	Adult mice	Zinc oxide nanoparticles Size: 30 nm	50 mg/kg/day 150 mg/kg/day 450 mg/kg/day	Oral exposure 14 consecutive days	Zinc testis concentration measured by AAS	No testicular distribution	Nil
Gaharwar et al., 2019 [143]	Adult rat	Iron oxide nanoparticles Size: 30 nm	7.5 mg/kg/w 15 mg/kg/w 30 mg/kg/w	Intravenous injection 4 consecutive weeks	Iron testis concentration measured by AAS	Low testicular distribution in comparison to other organs and only for the highest concentration	Between 1/10^4^ and 1/10^5^
Zhao et al., 2019 [144]	Adult mice	Food-borne nanoparticles Size: 5 to 8 nm	2 g/kg	Oral exposure. Single exposure with measurement for 30 min to 24 h	Fluorescence detection by a multifunctional in vivo imaging system.	Fluorescence detected in testis at all measurement times	Data not available
Nguyen et al., 2019 [145]	Adult mice	Cadmium telluride fluorescent quantum dots Size: 15 nm	0.4 to 10 mg/kg	Intravenous injection Single exposure with measurement from 2 h to 1 week after exposure	Cadmium testis concentration measured by ICP-MS. Fluorescence detection by spectrophoto-fluorometry.	Low testicular distribution in comparison to other organs	1/10^4^
Kielbik et al., 2019 [146]	Adult mice	Fluorescent europium-doped zinc oxide nanoparticles Size: 50 nm	3 mg/kg	Oral exposure Single exposure with measurement from 3 h to 2 weeks after exposure	Fluorescence detection in scanning microscopy	Testicular distribution with crossing of blood testis barrier	NA
Lopes et al., 2019 [147]	Prepubescent rats	Silver nanoparticles Size: 80 nm	1.875 to 15 µg/kg/2 days	Oral exposure From 23 days old to 60 days old	Silver testis concentration measured by AAS	Testicular biodistribution for 7.5 and 15 µg/kg/2 days doses	1/10^4^

**Table 2:** Summary of studies exploring the impact of nanoparticles on spermatic parameters. U: unchanged, no significant difference from control. A: altered, significant difference from control. NA: not available. MWCNT: multiwalled carbon nanotubes. SWCNT: single-walled carbon nanotubes. DSP: daily Sperm Production. h: hour. d: day. w: week.

Publication	Animal Model	Route of Exposure	Particle Model	Size (nm)	Particle Concentration	Duration of Exposure (Days)	Number of Sperm or DSP	Mobility	Morphology	DNA Fragmentation
Yoshida et al., 2009 [194]	Adult mice	Inhalation	Carbon black nanoparticles	14	0.1 mg/w	70	A	NA	NA	NA
				14	1.56 µg/w	70	A	NA	NA	NA
				56	0.1 mg/w	70	A	NA	NA	NA
				95	0.1 mg/w	70	A	NA	NA	NA
Bai et al., 2010 [113]	Adult mice	Intravenous	Carboxylate-functionalized MWCNT		0.1 mg/mouse or 5 mg/kg	5	U	U	U	U
Gromadzka-Ostrowska et al., 2012 [195]	Adult rats	Intravenous	Silver nanoparticles	20	5 mg/kg	1	A at 24 h & 28 d	NA	U	A at 24 h
					10 mg/kg	1	U	NA	U	A at 24 h
				200	5 mg/kg	1	U	NA	U	U
Li et al., 2012 [212]	Adult Mice	Inhalation	Diesel exhaust nanoparticles		41.73 μg/m^3^	40	U			
					152.01 μg/m^3^	40	U			
Wang et al., 2013 [123]	Adult rats	Intraarticular	Cobalt–chromium nanoparticles	55	20 µg/kg/w	70	U	U	U	NA
					100 µg/kg/w	70	U	U	U	NA
					500 µg/kg/w	70	A	A	A	NA
Xu et al., 2014 [197]	Adult mice	Intravenous	Silica nanoparticles	63	20 mg/kg/3 d	13	A at 15 d & 35 d	A at 15 d	A at 15 d & 35 d	A at 15 d & 35 d
Garcia et al., 2014 [124]	Adult mice	Intravenous	Citrate-coated silver nanoparticles	10	1 mg/kg/3 d	15	U	U	NA	NA
Castellini et al., 2014 [223]	Adult rabbits	Intravenous	Silver nanoparticles	45	0.6 mg/kg	1	A at 7 d & 21 d	A	A	NA
Jia et al., 2014 [198]	Adult mice	Oral	Titanium dioxide nanoparticles	25	10 mg/kg/d	42	U	NA	U	NA
					50 mg/kg/d	42	U	NA	A	NA
					250 mg/kg/d	42	U	NA	A	NA
Sleiman et al., 2014 and Mathais et al., 2015 [221,222]	Prepubescent rats	Oral	Silver nanoparticles	80	15 µg/kg/d	30–35	A	NA	A	NA
					30 or 50 µg/kg/d	30–35	A	NA	A	NA
Zhang et al., 2015 [187]	Prepubescent mice	Subcutaneous	Silver nanoparticles	15	1 mg/kg/3 d	13	U	NA	U	NA
					5 mg/kg/3 d		A	NA	A	NA
Wolterbeek et al., 2015 [231]	Adult rats	Oral	NM-200 synthetic amorphous silica	100	Up to 1000 mg/kg/d	70	U	U	U	NA
Abbasalipourkabir et al., 2015 [232]	Adult rats	Intraperitoneal	Zinc oxide nanoparticles	20	50 mg/kg/d	10	A	U	A	NA
					100 mg/kg/d	10	A	A	A	NA
					150 mg/kg/d	10	A	A	A	NA
					200 mg/kg/d	10	A	A	A	NA
Farcas et al., 2016 [216]	Adult mice	Inhalation	Cellulose nanocrystals	150	40 µg/d	6	A	A	A	A
Lafuente et al., 2016 [200]	Adult rats	Oral	Polyvinilpyrrolidone coated silver nanoparticles	20	50 mg/kg/d	90	U	U	U	NA
					100 mg/kg/d	90	U	U	A	NA
					200 mg/kg/d	90	U	U	A	NA
Li et al., 2016 [134]	Adult mice	Intravenous	Cadmium telluride fluorescent quantum dots	2	0.2 nmol	1	U	NA	U	U
					2 nmol	1	U	NA	A	A
Miura et al., 2017 [136]	Adult mice	Intravenous	Titanium dioxide nanoparticles	21	0.1 mg/kg/w	28	U	A	NA	NA
					1 mg/kg/w	28	U	A	NA	NA
					2 mg/kg/w	28	U	A	NA	NA
					10 mg/kg/w	28	A	A	NA	NA
Srivastav et al., 2017 [233]	Adult mice	Intraperitoneal	Zinc oxide nanoparticles	50	300 mg/kg/d	2	U	A	U	NA
					2000 mg/kg/d	2	A	A	A	NA
Nirmal et al., 2017 [202]	Adult rat	Intraperitoneal	Hydroxyl-functionalized MWCNT	20	0.4 mg/kg/2 d	30	U	U	U	NA
					2 mg/kg/2 d	30	A	U	A	NA
					10 mg/kg/2 d	30	A	A	A	NA
Nirmal et al., 2017 [201]	Adult rat	Intraperitoneal	Nano-graphene oxide	2 nm/10 µm	0.4 mg/kg/2 d	30	U	U	U	NA
					2 mg/kg/2 d	30	A at 31 d & U at d60	U	U	NA
					10 mg/kg/2 d	30	A at 31 d & U at d60	A at 31 d & U at d60	A at 31 d & U at d60	NA
Bai et al., 2018 [137]	Adult mice	Intraperitoneal	Tin sulfide nanoflowers	50	0.38 mg/kg/d	24	U	NA	NA	NA
				50	3.8 mg/kg/d	24	U	NA	NA	NA
				50	38 mg/kg/d	24	A	NA	NA	NA
				80	38 mg/kg/d	24	A	NA	NA	NA
				200	38 mg/kg/d	24	U	NA	NA	NA
Skovmand et al., 2018 [203]	Adult mice	Inhalation	Graphene oxide	2–3 µm	0.1 mg/w	49	U	U	U	U
			Flammruss 101 (carbon black)	95	0.1 mg/w	49	U	U	U	U
			Printex 90 (carbon black)	14	0.1 mg/w	49	U	U	U	U
			SRM 1650b (Diesel exhaust)	18–30	0.1 mg/w	49	U	U	U	U
Fang et al., 2018 [235]	Adult mice	Oral	MWCNT		100 mg/kg/d	5	NA	NA	NA	A
Aabi et al., 2019 [234]	Adult mice	Intraperitoneal	Solid lipid nanoparticles	142	170 µg/kg/d	5	U	NA	A	NA
			Superparamagnetic iron oxide nanoparticles	112	5 mg/kg/d	5	U	NA	A	NA
			Both nanoparticles		Same	5	A	NA	A	NA
Skovmand et al., 2020 [219]	Adult rat	Inhalation	Welding fumes particles	NA	20 mg/m^3^ 3 h/d 4 d/w	5 w	A	NA	NA	NA
Tang et al., 2020 [142]	Adult mice	Oral	Zinc oxide nanoparticles	30	50 mg/kg/d	14	U	NA	NA	NA
					150 mg/kg/d	14	A	NA	NA	NA
					450 mg/kg/d	14	A	NA	NA	NA
Kielbik et al., 2019 [146]	Adult mice	Oral	Fluorescent europium-doped zinc oxide nanoparticles	50	3 mg/kg	1	U	Slight increase	NA	NA
Lauvås et al., 2019 [220]	Adult mice	Inhalation	Titanium dioxide nanoparticles	17	63 µg/w	49	U	NA	NA	NA
Mohammadi et al., 2020 [236]	Adult mice	Intravenous	COOH SWCNT		4 mg/kg/w	35	A	A	U	NA
			COOH MWCNT		4 mg/kg/w	35	A	A	A	NA
			NH_2_ SWCNT		4 mg/kg/w	35	A	A	A	NA
			NH_2_ MWCNT		4 mg/kg/w	35	A	A	A	NA

## Data Availability

Not applicable.
